# Correction: Rates and risk factors for antepartum and intrapartum stillbirths in 20 secondary hospitals in Imo state, Nigeria: A hospital-based case control study

**DOI:** 10.1371/journal.pgph.0005056

**Published:** 2025-08-12

**Authors:** The PLOS One Staff

## Notice of republication

This article was republished on July 16, 2025, to correct an error in the sixth author’s name. The correct name is: Manisha Nair. The correct citation is: Gwacham-Anisiobi U, Opondo C, Cheng TS, Kurinczuk JJ, Anyaegbu G, Nair M (2024) Rates and risk factors for antepartum and intrapartum stillbirths in 20 secondary hospitals in Imo state, Nigeria: A hospital-based case control study. PLOS Glob Public Health. 4(10): e0003771. https://doi.org/10.1371/journal.pgph.0003771

The copyright statement for this article has also been updated to include the corrected authors name.

## Supporting information

S1 File Originally published, uncorrected article. (PDF)

S2 File Republished, corrected article. (PDF)
