# Supplementary material for: Rates and risk factors for antepartum and intrapartum stillbirths in 20 secondary hospitals in Imo state, Nigeria: A hospital-based case control study
Source: PLOS Glob Public Health. 2024 Oct 24;4(10):e0003771. doi: 10.1371/journal.pgph.0003771 (PMC11500848; doi:10.1371/journal.pgph.0003771)
Supplement: S1 Table — (PDF) [file pgph.0003771.s001.pdf]

Study sample derivation

A sample size of 146 cases each for antepartum and intrapartum stillbirths and 292 controls using 1 case:2 controls was calculated for antepartum and intrapartum stillbirths respectively using the Kelsey equation

S1 Table: Sample size calculation using Kelsey equation

				**Number of cases required to detect the effect size (Kelsey equation)**				
**Prevalence of risk factor in controls**	**case: control ratio**	**power**	**CI**	**1.5**	**1.6**	**1.8**	**1.85**	**2.0**
0.05	1:2	80	95%	1193	857	515	463	351
0.10	1:2	80	95%	675	469	285	257	151
0.15	1:2	80	95%	447	343	210	190	146
0.20	1:2	80	95%	388	283	175	159	123
0.25	1:2	80	95%	341	250	156	142	110
