# Supplementary material for: Rates and risk factors for antepartum and intrapartum stillbirths in 20 secondary hospitals in Imo state, Nigeria: A hospital-based case control study
Source: PLOS Glob Public Health. 2024 Oct 24;4(10):e0003771. doi: 10.1371/journal.pgph.0003771 (PMC11500848; doi:10.1371/journal.pgph.0003771)
Supplement: S2 Table — (PDF) [file pgph.0003771.s002.pdf]

To account for clustering, the design effect was calculated for a range of intraclass correlation coefficients (ICC) and the sample size was inflated further by 10% to account for missing data and confounding. An ICC of 0.001 was considered as most pragmatic for this project. Thus, the total minimum sample size required was 692 – disaggregated into 173 cases of antepartum stillbirths, 173 cases of intrapartum stillbirths and 346 controls.

S2 Table: Design effect and missing data inflation calculation

	**Design effect (based on total sample size of 584)**		**Adjusted sample size** (based on an ICC of 0.001)		**10% inflation for missing data**	
Intraclass Correlation coefficient	15 hospitals	20 hospitals	15 hospitals	20 hospitals	15 hospitals	20 hospitals
0.001	1.038	1.028	606	600	667	692
0.01	1.38	1.28	806	748	1465	823
0.05	2.9	2.41	1694	1407	1950	1548
