# Supplementary material for: Rates and risk factors for antepartum and intrapartum stillbirths in 20 secondary hospitals in Imo state, Nigeria: A hospital-based case control study
Source: PLOS Glob Public Health. 2024 Oct 24;4(10):e0003771. doi: 10.1371/journal.pgph.0003771 (PMC11500848; doi:10.1371/journal.pgph.0003771)
Supplement: S3 Table — (PDF) [file pgph.0003771.s003.pdf]

S3 Table: Health facilities included in the study and their characteristics

| Health facility ID | Ownership | Local Government Area | Zone |
|---|---|---|---|
| 1 | Government | Aboh Mbaise | Owerri |
| 2 | Government | Oru East | Owerri |
| 3 | Mission | Nwangele | Orlu |
| 4 | Mission | Isu | Orlu |
| 5 | Mission | Owerri North | Owerri |
| 6 | Mission | Aboh Mbaise | Owerri |
| 7 | Private | Aboh Mbaise | Owerri |
| 8 | Private | Owerri North | Owerri |
| 9 | Private | Orlu | Orlu |
| 10 | Private | Owerri Municipal | Owerri |
| 11 | Mission | Owerri Municipal | Owerri |
| 12 | Private | Mbaitoli | Owerri |
| 13 | private | Owerri North | Owerri |
| 14 | private | Ahiazu Mbaise | Owerri |
| 15 | Private | Owerri Municipal | Owerri |
| 16 | Private | Owerri Municipal | Owerri |
| 17 | Government | Owerri west | Owerri |
| 18 | Private | Owerri North | Owerri |
| 19 | Private | Owerri west | Owerri |
| 20 | Private | Owerri Municipal | Owerri |

LGA- Local Government Area
