# Supplementary material for: Rates and risk factors for antepartum and intrapartum stillbirths in 20 secondary hospitals in Imo state, Nigeria: A hospital-based case control study
Source: PLOS Glob Public Health. 2024 Oct 24;4(10):e0003771. doi: 10.1371/journal.pgph.0003771 (PMC11500848; doi:10.1371/journal.pgph.0003771)
Supplement: S4 Table — (PDF) [file pgph.0003771.s004.pdf]

S4 Table: Univariable analysis of risk factors associated with antepartum and intrapartum stillbirths

Variable	**Antepartum SB** Crude OR(95% CI)	p-Value	**Intrapartum SB** Crude OR(95% CI)	p-Value
**Sociodemographic factors**				
**Maternal age**	1.03 (1.00-1.07)	0.06	1.04 (1.00 – 1.07)	0.02
**Marital status**<br>Married<br>Not married	1 (ref)<br>1.65 (0.56 – 4.71)	0.35	1 (ref)<br>3.19 (1.33- 7.61)	0.01
**Place of residence**<br>Rural<br>Urban	1 (ref)<br>0.85 (0.58 – 1.26)	0.43	1 (ref)<br>0.77 (0.53 – 1.11)	0.17
**Intermediate factors**				
**Parity**<br>0<br>1-3<br>4 or more	1.39 (0.93 - 2.08))<br>1 (ref)<br>1.90 (1.0 - 3.64)	0.06	1.38 (0.95 - 2.02)<br>1 (ref)<br>2.58 (1.45 – 4.59)	0.004
**Stillbirth history**<br>No<br>Yes	1 (ref)<br>1.13 (0.57 – 2.24)	0.74	1 (ref)<br>0.93 (0.48 – 1.81)	0.83
**Gestational age**<br>Preterm<br>Term<br>Late term	13.20 (7.22 - 24.16)<br>1 (ref)<br>1.71 (0.83 - 3.5)	<0.001	9.02 (4.98 - 16.38)<br>1 (ref)<br>1.31 (0.66-2.62)	<0.001
**Number of ANC visits**<br>Unbooked<br>1-3 visits<br>4 or more visits	5.98(3.84 - 9.30)<br>0.72 (0.48 – 4.92)<br>1(ref)	<0.001	3.51 (2.32 - 5.32)<br>2.14 (0.87– 5.23)<br>1 (ref)	<0.001
Referral status<br>No<br>Yes	1 (ref)<br>6.04 (3.91 - 9.32)	<0.001	1 (ref)<br>4.31(2.83 – 6.53)	<0.001
**Past obstetric complication** (all)<br>No<br>Yes	1 (ref)<br>1.15 (0.59 – 2.22)	0.69	1 (ref)<br>0.71 (0.35 – 1.44)	0.34
**Type of facility of birth**<br>Public<br>Private<br>Mission	1 (ref)<br>0.73(0.46- 1.14)<br>0.68 (0.42 – 1.09)	0.23	1 (ref)<br>1.08 (0.69 – 1.71)<br>1.45 (0.92 – 2.27)	0.20
**Mode of childbirth**<br>Vaginal birth<br>Caesarean birth	-<br>-	-	1 (ref)<br>2.31(1.60 – 3.32)	<0.001
**Proximal factors**				

**Medical comorbidities** (all)<br>No<br>Yes	1 (ref)<br>3.85 (1.55 – 9.63)	0.004	1 (ref)<br>1.50 (0.51 – 4.37)	0.46
**One or more Obstetric complications in the index pregnancy**<br>No<br>Yes	1 (ref)<br>7.34 (4.29 – 12.54)	<0.001	1(ref)<br>10.21 (6.81 – 15.30)	<0.001
**Antepartum haemorrhage**<br>No<br>Yes	1 (ref)<br>4.4 (1.87 – 10.20)	0.001	1 (ref)<br>7.91 (3.68 – 16.99)	<0.001
**Hypertensive disease of pregnancy**<br>No<br>Yes	1 (ref)<br>7.52 (3.92 – 14.45)	<0.001	1 (ref)<br>3.55 (1.78 – 0.06)	<0.001
**Abnormal presentation**<br>No<br>Yes	-	-	1(ref)<br>11.19 (4.21 - 29.73)	<0.001
**Malaria in pregnancy**<br>No<br>Yes	1 (ref)<br>2.47 (0.71 – 8.67)	0.12	1<br>3.68 (1.22 – 11.13)	0.02
**Obstructed labour**<br>No<br>Yes	-<br>-	-	1 (ref)<br>2.74(1.72 - 4.38)	<0.001
**Fetal factors**				
Sex<br>Male<br>Female	1 (ref)<br>0.71 (0.48 – 1.06)	0.52	1 (ref)<br>0.82 (0.57 – 1.18)	0.28
**Use of partogram in labour**<br>No<br>Yes<br>Not Indicated	-<br>-	-	2.13 (1.21 - 3.77)<br>1 (ref)<br>4.19 (2.19 - 8.03)	0.0001
