# Supplementary material for: Rates and risk factors for antepartum and intrapartum stillbirths in 20 secondary hospitals in Imo state, Nigeria: A hospital-based case control study
Source: PLOS Glob Public Health. 2024 Oct 24;4(10):e0003771. doi: 10.1371/journal.pgph.0003771 (PMC11500848; doi:10.1371/journal.pgph.0003771)
Supplement: S5 Table — (PDF) [file pgph.0003771.s005.pdf]

S5 Table: Comparison between complete case and multiple imputation models for antepartum stillbirths

Complete case analysis- (447)				Multiple imputation by chained equation – MICE - 519			
Parameter	OR	SE	P val	Parameter	OR	SE	P val
Intercept	0.02	0.02	<0.001	Intercept	0.03	0.02	<0.001
Maternal age	1.04	0.03	0.20	Maternal age	1.03	0.03	0.263
**Gest. age**<br>Term<br>preterm	 1<br>13.62	 <br>5.63	 <br><0.001	**Gest. age**<br>Term<br>preterm	 1<br>12.21	 -<br>4.48	 <br><0.001
Parity<br>0<br>1-3<br>4 or more	 1.84<br>1<br>1.25	 0.55<br>-<br>0.61	 <br>0.12	Parity<br>0<br>1-3<br>4 or more	 1.57<br>1<br>1.17	 0.44<br>-<br>0.53	 0.28
**Number of ANC visits**<br>Unbooked<br>1-3 visits<br>4 or more visits	 2.09<br>0.92<br>1	 0.70<br>0.71<br>-	 <br>0.08	**Number of ANC visits**<br>Unbooked<br>1-3 visits<br>4 or more visits	 2.26<br>1.13<br>1	 0.72<br>0.85<br>-	0.05
Referral status	3.09	1.05	0.001	Referral status	2.94	0.93	0.001
Medical comorbidity	1.94	1.21	0.29	Medical comorbidity	2.21	1.27	0.17
Pregnancy complication	4.06	1.12	<0.001	Pregnancy complication	4.03	1.01	<0.001
