# Supplementary material for: Rates and risk factors for antepartum and intrapartum stillbirths in 20 secondary hospitals in Imo state, Nigeria: A hospital-based case control study
Source: PLOS Glob Public Health. 2024 Oct 24;4(10):e0003771. doi: 10.1371/journal.pgph.0003771 (PMC11500848; doi:10.1371/journal.pgph.0003771)
Supplement: S6 Table — (PDF) [file pgph.0003771.s006.pdf]

S6 Table: Comparison between complete case and multiple imputation models for intrapartum stillbirths

Complete case analysis (453)				Multiple imputation by chained equation – MICE (508)			
Parameter	OR	SE	P val	Parameter	OR	SE	P val
Intercept	0.01	0.01	<0.001	Intercept	0.01	0.01	<0.001
Maternal age	1.03	0.26	0.19	Maternal age	1.05	0.06	0.04
Marital status<br>Married<br>Not married	 1<br>2.79	 -<br>1.93	 <br>0.14	Marital status<br>Married<br>Not married	 1<br>3.74	 <br>2.41	 <br>0.04
**Gest. age**<br>Term<br>preterm	 1<br>11.08	 -<br>4.93	 <br><0.001	**Gest. age**<br>Term<br>preterm	 1<br>8.29	 -<br>3.31	 <br><0.001
**Number of ANC visits**<br>Unbooked<br>1-3 visits<br>4 or more visits	 1.42<br>1.40<br>1	 0.53<br>0.88<br>-	 <br>0.58	**Number of ANC visits**<br>Unbooked<br>1-3 visits<br>4 or more	 1.35<br>1.62<br>1	 0.47<br>0.97<br>-	 0.53
Mode of childbirth<br>Vaginal<br>Caesarean	 <br>1<br>0.69	 <br>-<br>0.23	 <br><br>0.27	Mode of childbirth<br>Vaginal<br>Caesarean	 <br>1<br>0.69	 <br><br>0.22	 <br><br>0.241
Referral status	2.33	0.87	0.02	Referral status	2.71	0.96	0.01
Partogram use<br>Yes<br>No<br>Not indicated	 1<br>3.02<br>3.91	 -<br>1.33<br>2.09	 <br>0.03	Partogram use<br>Yes<br>No<br>Not indicated	 1<br>2.56<br>3.01	 <br>1.05<br>1.49	 <br>0.05
Pregnancy complication	11.17	3.34	<0.001	Pregnancy complication	11.34	3.22	<0.001
