# Supplementary material for: Rates and risk factors for antepartum and intrapartum stillbirths in 20 secondary hospitals in Imo state, Nigeria: A hospital-based case control study
Source: PLOS Glob Public Health. 2024 Oct 24;4(10):e0003771. doi: 10.1371/journal.pgph.0003771 (PMC11500848; doi:10.1371/journal.pgph.0003771)
Supplement: S7 Table — (PDF) [file pgph.0003771.s007.pdf]

S7 Table: Effect of obstetric complications on stillbirths after adjusting for sociodemographic and intermediate factors.

**Complication**	**Antepartum stillbirths (n = 157)**				**Intrapartum stillbirths (n= 193)**			
	n (%)	Crude OR (95% CI)	AOR (95% CI)	**PAF** (95% CI)	n (%)	Crude OR	AOR	PAF (95% CI)
Prolonged labour	24 (15.3)	1.58 (0.92 – 2.73)	1.63 (0.73 – 3.54)	5.8% (1.6 – 12.6)	46 (23.8)	2.74(1.71 – 4.38)	6.85(3.23 – 14.50)	20% (17.4 – 22.5)
Malaria	5 (3.2)	2.47 (0.71 – 8.67)	1.34 (0.22 – 8.18)	0.8% (-3.4 – 4.9)	9 (4.6)	3.68 (1.22 – 11.13)	4.73 (1.17 - 19.16)	3.8% (2.4 – 5.2)
Premature Rupture of membranes	16 (10.2)	3.82 (1.73 – 8.43)	1.13 (0.23 – 5.51)	0.9% (- 10.8 – 11.3)	22 (11.4)	4.33 (2.05 –9.13)	4.84 (1.46-16.06)	9.8% (6.7 – 12.9)
Abnormal presentation	14 (8.9)	7.36 (2.60 – 20.81)	7.80 (2.20 – 27.67)	6.9% (5.6 – 8.1)	25 (13.0)	11.19 (4.21 – 29.73)	17.14 (5.03 – 58.38)	13.0% (12.0 – 14.0)
Uterine rupture	4(2.5)	3.29 (0.73– 14.89)	2.84 (0.43 – 18.98)	6.9% (5.6 – 8.1)	13 (6.7)	9.1 (2.56 –32.33)	8.32 (1.75 – 39.57)	1.5% (- 0.06 – 3.1)
Antepartum Haemorrhage	15(9.6)	3.63 (1.12 – 11.73	5.85 (1.74-19.60)	7.8% (5.9 – 9.8)	31 (16.1)	7.91 (3.68-16.99)	17.75 (5.60 - 56.27)	15.6% (14.5 – 16.7)
Hypertensive disorders of Preg.	35 (22.3)	7.52 (3.92 – 14.45)	14.20(5.76 – 34.99)	21.3%	23(11.9)	3.54 (1.78 – 10.06)	8.21 (3.08 – 21.89)	12.1% (10.4 – 13.7)
