# Supplementary figures and images for: Rates and risk factors for antepartum and intrapartum stillbirths in 20 secondary hospitals in Imo state, Nigeria: A hospital-based case control study
Source: PLOS Glob Public Health. 2024 Oct 24;4(10):e0003771. doi: 10.1371/journal.pgph.0003771 (PMC11500848; doi:10.1371/journal.pgph.0003771)

S1 Fig : Flowchart for selection of study sample included in the analysis

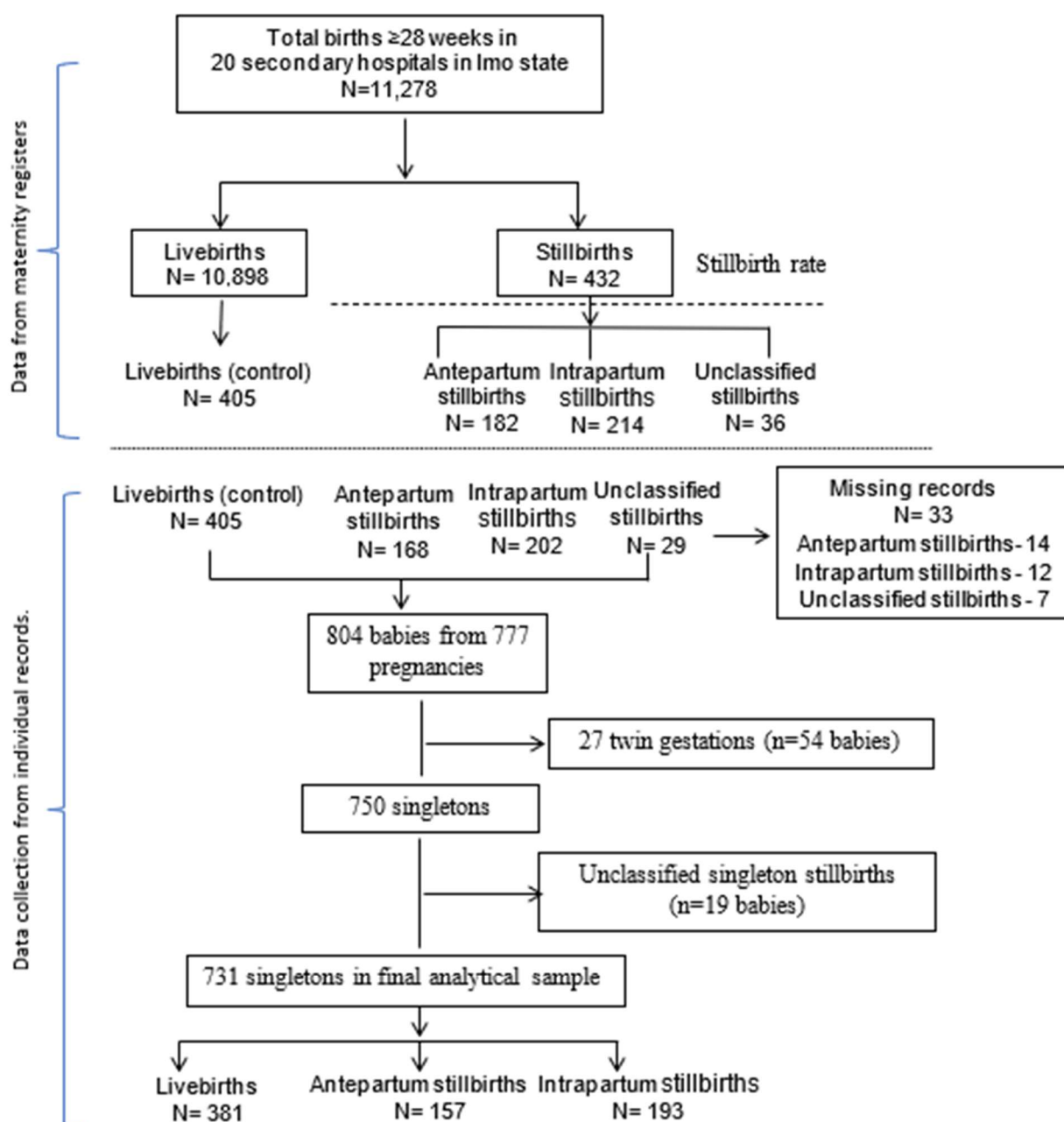

Supplement: S1 Fig — (PDF) [file pgph.0003771.s008.pdf]
