# Supplementary material for: Rates and risk factors for antepartum and intrapartum stillbirths in 20 secondary hospitals in Imo state, Nigeria: A hospital-based case control study
Source: PLOS Glob Public Health. 2024 Oct 24;4(10):e0003771. doi: 10.1371/journal.pgph.0003771 (PMC11500848; doi:10.1371/journal.pgph.0003771)
Supplement: Attachment — Submitted filename: Paper review SB.docx [file pgph.0003771.s009.docx]

The paper has put in context how much still birth has been a daunting problem in Nigeria and Imo state and the little progress over the years. It has also laid out what the important sociodemographic, obstetric and maternity and biological factors are at play for still birth. Looked at the important determinants for both ASB and ISB through a case control study and shade light on the important related factors such as obstetric complication, use of partograph, booking status and delayed referral with recommendation to address such issues and policy implications

The study was a retrospective study and there has been selection of facilities based on access on security status which again might have impact on the results as we expect cases coming late in the difficult to access facilities with potentially high rates of still birth.

Whereas the data in the relatively accessible might have given better outcome it may as well be showing a false kick start to progress

On the same point lots of exclusion due to missing data might also be impacting the interpretation of results, as such although this has been in some form stated in the discussion probably both facility selection and data exclusion have to come bold as limitation of the study

Why did you not put effort to match the cases and controls?

What is the result from your study on ISB versus mode of delivery (SVD, assisted vaginal birth and C/S)?

Do you think including findings on data completeness and recommendation on how to improving data for better tracking will be of help

Any recommendations on study approach which you will like to follow up on your effort, especially in light of addressing the short falls your study pointed?

Any points you would like to make on the unclassified cases in your study compared to findings elsewhere?

Much of the references on your discussion is on local studies within Nigeria for major part how does your study finding compare with finding from similar studies of your kind elsewhere? do you see is important if you could expand a bit on this?
