# Supplementary material for: Rates and risk factors for antepartum and intrapartum stillbirths in 20 secondary hospitals in Imo state, Nigeria: A hospital-based case control study
Source: PLOS Glob Public Health. 2024 Oct 24;4(10):e0003771. doi: 10.1371/journal.pgph.0003771 (PMC11500848; doi:10.1371/journal.pgph.0003771)
Supplement: Attachment — Submitted filename: revision_response to reviewers_final_290624.docx [file pgph.0003771.s010.docx]

23^rd^ June, 2024

Dr. Catherine Kyobutungi

Editor-in-Chief

PLOS Global Public Health

Dear Editor,

**Re: Rates and risk factors for antepartum and intrapartum stillbirths in 20 secondary hospitals in Imo state, Nigeria: a hospital-based case-control study.**

Thank you very much for considering our paper and for the editorial and reviewer which have helped us to significantly improve the quality of our  paper.

We have addressed these comments as tracked changes in the manuscript and provided point-by-point responses as set out below.

We look forward to your feedback.

Thank you for your consideration.

Regards,

Dr Uchenna Gwacham-Anisiobi

Corresponding author

on behalf of all authors

**Updated comments to Authors**

1. Please provide additional details regarding participant consent. In the ethics statement in the Methods and online submission information, please ensure that you have specified (1) whether consent was informed and (2) what type you obtained (for instance, written or verbal, and if verbal, how it was documented and witnessed). If your study included minors, state whether you obtained consent from parents or guardians. If the need for consent was waived by the ethics committee, please include this information.

If you are reporting a retrospective study of medical records or archived samples, please ensure that you have discussed whether all data were fully anonymized before you accessed them and/or whether the IRB or ethics committee waived the requirement for informed consent. If patients provided informed written consent to have data from their medical records used in research, please include this information.

**Response:** Thank you for pointing out this oversight in the last response document. This information has now been included in the methods section of the manuscript.

“*Ethics approval was obtained from Federal Medical Centre, Owerri (FMC/OW/HREC/VOL.II/68), Imo State Specialist Hospital and Oxford Tropical Research Ethics Committees (OxTREC Ref: 535-22). Both ethics committees waived the requirement for informed consent. Only UGA and the research midwives in each facility had access to the identifiable medical records at the point of data collection in each facility. Each participant’s data were anonymised at point of collection making it impossible to trace data back to participants after daily validation. The rest of the research team accessed only fully anonymised data for the analysis. There was no direct contact with participants for this study*.” Page 6, Line 230 - 236

**COMMENTS TO THE AUTHOR:**

**Reviewer #1**: The paper has put in context how much still birth has been a daunting problem in Nigeria and Imo state and the little progress over the years. It has also laid out what the important sociodemographic, obstetric and maternity and biological factors are at play for still birth. Looked at the important determinants for both ASB and ISB through a case control study and shade light on the important related factors such as obstetric complication, use of partograph, booking status and delayed referral with recommendation to address such issues and policy implications

**Response:**  We are grateful to the reviewer for this insightful summary, the acknowledgement of the risk factors explored and the implications of the study findings for policy and practice. Thank you.

**Reviewer #1**: The study was a retrospective study and there has been selection of facilities based on access on security status which again might have impact on the results as we expect cases coming late in the difficult to access facilities with potentially high rates of still birth.

Whereas the data in the relatively accessible might have given better outcome it may as well be showing a false kick start to progress

**Response:**  Thank you for this feedback. The hospital selection for this study is an important limitation which should be highlighted in as you have rightly mentioned has implications for the data. We have included the following lines in the discussion to highlight that:

*“we acknowledge important limitations in our study. Our hospital selection was limited to LGAs considered safe at the point of data collection introducing potential selection bias and affecting the generalisability of our findings.* *A consequence of the potential selection bias would be an underestimation or overestimation of stillbirth incidence.* Page 17 line 418-421

**Reviewer #1**: On the same point lots of exclusion due to missing data might also be impacting the interpretation of results, as such although this has been in some form stated in the discussion probably both facility selection and data exclusion have to come bold as limitation of the study

**Response:**  Thank you for this feedback. Yes we agree that the exclusion of data for important variables especially those which have been found important in other studies is an important limitation for this study. We have included the following in the limitations:

*“Secondly, important variables such as fetal weight for gestational age, socioeconomic status, presence of fetal distress amongst others were excluded from our analysis due to large proportions of missing data, thus, we cannot completely rule out the possibility of residual confounding.”* Page 17 line 423-424

**Reviewer #1**: Why did you not put effort to match the cases and controls?

**Response:** This is because of our research question. We aimed to understand the risk factors associated with intrapartum and antepartum stillbirths generally. Our research questions were exploratory in nature and not hypothesis-driven, meaning they were not conditioned on any specific factor. Therefore, it was not appropriate to match the cases and controls.^1^

**Reviewer #1**: What is the result from your study on ISB versus mode of delivery (SVD, assisted vaginal birth and C/S)?

**Response:**  Thank you for this question. We planned to analyse the association of the mode of childbirth (SVD, assisted vaginal birth, elective and emergency CS) with intrapartum stillbirth. However, we had very few cases of assisted vaginal births (5 in cases, 3 in controls) and elective CS (2 in cases, 36 in controls). These numbers were too small for a robust analysis and including these small numbers in a regression model would have led to unreliable estimates and potentially inflated standard errors. Therefore, we decided to group all vaginal births together and all caesarean births together to maintain the statistical validity of our analysis.

We have included this information in the methods section (page 5 Line 206 - 211)

**Reviewer #1**: Do you think including findings on data completeness and recommendation on how to improving data for better tracking will be of help

**Response:**  Thank you for this question. We briefly highlighted the relevance of data completeness for epidemiological studies in the discussion but we have now expanded it to include your feedback.

*It is imperative to establish robust systems for classifying and reporting stillbirths. Improving data completeness and reporting requires periodic training for health workers and strengthening data collection systems infrastructure to ensure accurate and consistent reporting. A shift to digital health records could help streamline data collection, potentially reduce errors and improve timely access to stillbirth data. However, the implementation of electronic medical records in Nigeria has been fraught with many challenges, including poor infrastructure, funding constraints and resistance by health workers.^2^* (page 17 Line 431 - 436)

**Reviewer #1**: Any recommendations on study approach which you will like to follow up on your effort, especially in light of addressing the short falls your study pointed?

**Response:**  Thank you for pointing this out. We have included the following line:

*“**Future research should aim for more rigorous designs, to address these limitations. This study serves as a baseline on which other hypothesis driven studies could be built.”* Page 17, line 429-430

**Reviewer #1**: Any points you would like to make on the unclassified cases in your study compared to findings elsewhere?

**Response:**  Thank you for flagging this. We have clarified the use of the term ‘unclassified’ stillbirths in our study in comparison to other studies where the term ‘unclassified stillbirths’ may be used. The following text has been added:

“*The term ‘unclassified’ in this study denotes instances where the stillbirth type is undocumented in maternal register and either the medical records were unavailable for review or had insufficient information to decipher the type of stillbirth. This definition differs from its use in Relevant Condition at Death (ReCoDe) classification,* *which is a system used to classify the underlying cause of death in perinatal death and takes into account both maternal and fetal conditions to determine the primary cause of death .*^3^*”* (page 7 – line 252-257)

**Reviewer #1**: Much of the references on your discussion is on local studies within Nigeria for major part how does your study finding compare with finding from similar studies of your kind elsewhere? do you see is important if you could expand a bit on this?

**Response:**  Thank you for your feedback. We have included additional comparisons with studies conducted in other Sub-Saharan African contexts, other low- and middle-income countries, and high-income countries for a more comprehensive analysis. The studies cited have been included into the updated reference list

*These challenges are not unique to Nigeria and were reported in a systematic reviews including other countries in Africa ^4^ and another review from India.^5^* (page 16 Line 375 - 376)

*In contrast, a systematic review and metanalysis from sub-saharan Africa^6^ and specific study in Ethiopia^7^ showed that nulliparity was protective for stillbirth. It is worth investigating further into reasons for the increased stillbirth risk for nulliparous women in Nigeria in comparison with other African countries.* (page 16 Line 381 - 390)

**Other editorial comments:**

Dear Dr. Gwacham-Anisiobi,

Thank you for submitting your manuscript to PLOS Global Public Health. After careful consideration, we feel that it has merit but does not fully meet PLOS Global Public Health’s publication criteria as it currently stands. Therefore, we invite you to submit a revised version of the manuscript that addresses the points raised during the review process.

**Response:**  Thank you for considering our paper and the invitation to submit a revised version of the manuscript.

Please note that we have only been able to secure a single reviewer to assess your manuscript. We are issuing a decision on your manuscript at this point to prevent further delays in the evaluation of your manuscript. Please be aware that the editor who handles your revised manuscript might find it necessary to invite additional reviewers to assess this work once the revised manuscript is submitted. However, we will aim to proceed on the basis of this single review if possible.

**Response:**  Thank you for the information. We appreciate the effort to prevent further delays in the evaluation of our manuscript. We have noted the points raised by the reviewer and have addressed them thoroughly in our revision. We understand that additional reviewers may be invited for further assessment if deemed necessary, and we are prepared to respond to any additional feedback.

Please see the comments form the reviewer in the attached document. Could you please revise the manuscript to carefully address the concerns raised?

**Response:**  Thank you. All comments raised have been addressed in the revised version uploaded.

**Journal Requirements:**

1. Please review your reference list to ensure that it is complete and correct. If you have cited papers that have been retracted, please include the rationale for doing so in the manuscript text, or remove these references and replace them with relevant current references. Any changes to the reference list should be mentioned in the rebuttal letter that accompanies your revised manuscript. If you need to cite a retracted article, indicate the article’s retracted status in the References list and also include a citation and full reference for the retraction notice.

**Response:**  Thank you. We have not cited any retracted papers. However, to fully address the comments of the reviewer, we have included six additional references to the reference list.

2. Please provide additional details regarding participant consent. In the ethics statement in the Methods and online submission information, please ensure that you have specified (1) whether consent was informed and (2) what type you obtained (for instance, written or verbal, and if verbal, how it was documented and witnessed). If your study included minors, state whether you obtained consent from parents or guardians. If the need for consent was waived by the ethics committee, please include this information.

If you are reporting a retrospective study of medical records or archived samples, please ensure that you have discussed whether all data were fully anonymized before you accessed them and/or whether the IRB or ethics committee waived the requirement for informed consent. If patients provided informed written consent to have data from their medical records used in research, please include this information.

**Response:**  Thank you. Our study is a retrospective study using medical records. The local and University of Oxford ethics committees waived the requirement for informed consent for this study. Only UGA and the research midwives in each facility had access to the identifiable medical records at the point of data collection in each facility. Each participant’s data were anonymised at point of collection making it impossible to trace data back to participants after daily validation. The rest of the research team accessed only fully anonymised data for the analysis. There was no direct contact with participants for this study. These details have been included in the relevant section of the manuscript.

3. Please provide separate figure files in .tif or .eps format only and remove any figures embedded in your manuscript file. Please also ensure all files are under our size limit of 10MB.

For more information about figure files please see our guidelines:

<https://journals.plos.org/globalpublichealth/s/figures> 
<https://journals.plos.org/globalpublichealth/s/figures#loc-file-requirement>

**Response:**  Thank you. All figures have now been provided in the .tif format and removed from the manuscript file.

4. We have noticed that you have uploaded Supporting Information files, but you have not included a list of legends. Please add a full list of legends for your Supporting Information files after the references list.

**Response:**  Thank you. A full list of legends for the supporting information file has now been included after the reference list.

5. Some material included in your submission may be copyrighted. According to PLOS’s copyright policy, authors who use figures or other material (e.g., graphics, clipart, maps) from another author or copyright holder must demonstrate or obtain permission to publish this material under the Creative Commons Attribution 4.0 International (CC BY 4.0) License used by PLOS journals. Please closely review the details of PLOS’s copyright requirements here: PLOS Licenses and Copyright. If you need to request permissions from a copyright holder, you may use PLOS's Copyright Content Permission form.

Please respond directly to this email or email the journal office and provide any known details concerning your material's license terms and permissions required for reuse, even if you have not yet obtained copyright permissions or are unsure of your material's copyright compatibility.

Potential Copyright Issues:

Fig 2: please (a) provide a direct link to the base layer of the map (i.e., the country or region border shape) and ensure this is also included in the figure legend; and (b) provide a link to the terms of use / license information for the base layer image or shapefile. We cannot publish proprietary or copyrighted maps (e.g. Google Maps, Mapquest) and the terms of use for your map base layer must be compatible with our CC-BY 4.0 license.

Note: if you created the map in a software program like R or ArcGIS, please locate and indicate the source of the basemap shapefile onto which data has been plotted.

If your map was obtained from a copyrighted source please amend the figure so that the base map used is from an openly available source. Alternatively, please provide explicit written permission from the copyright holder granting you the right to publish the material under our CC-BY 4.0 license.

Please note that the following CC BY licenses are compatible with PLOS license: CC BY 4.0, CC BY 2.0 and CC BY 3.0, meanwhile such licenses as CC BY-ND 3.0 and others are not compatible due to additional restrictions.

If you are unsure whether you can use a map or not, please do reach out and we will be able to help you. The following websites are good examples of where you can source open access or public domain maps: 
* U.S. Geological Survey (USGS) - All maps are in the public domain. ([http://www.usgs.gov](http://www.usgs.gov/)) 
* PlaniGlobe - All maps are published under a Creative Commons license so please cite “PlaniGlobe, [http://www.planiglobe.com](http://www.planiglobe.com/), CC BY 2.0” in the image credit after the caption. (<http://www.planiglobe.com/?lang=enl>) 
* Natural Earth - All maps are public domain. (<http://www.naturalearthdata.com/about/terms-of-use/>)

**Response:**  Thank you for the feedback. A new map plotted on ArcGIS by one of the authors using a basemap shapefile which has a CC-BY 4.0 license has been uploaded in place of the previous file. The file has been adequately referenced.

Additional Editor Comments (if provided):


[Note: HTML markup is below. Please do not edit.]

Reviewers' comments:

Reviewer's Responses to Questions

**Comments to the Author**

1. Does this manuscript meet PLOS Global Public Health’s [publication criteria](https://journals.plos.org/globalpublichealth/s/criteria-for-publication)? Is the manuscript technically sound, and do the data support the conclusions? The manuscript must describe methodologically and ethically rigorous research with conclusions that are appropriately drawn based on the data presented.

Reviewer #1: Yes

2. Has the statistical analysis been performed appropriately and rigorously?

Reviewer #1: I don't know

3. Have the authors made all data underlying the findings in their manuscript fully available (please refer to the Data Availability Statement at the start of the manuscript PDF file)?

The [PLOS Data policy](https://journals.plos.org/globalpublichealth/s/data-availability) requires authors to make all data underlying the findings described in their manuscript fully available without restriction, with rare exception. The data should be provided as part of the manuscript or its supporting information, or deposited to a public repository. For example, in addition to summary statistics, the data points behind means, medians and variance measures should be available. If there are restrictions on publicly sharing data—e.g. participant privacy or use of data from a third party—those must be specified.

Reviewer #1: No

4. Is the manuscript presented in an intelligible fashion and written in standard English?

PLOS Global Public Health does not copyedit accepted manuscripts, so the language in submitted articles must be clear, correct, and unambiguous. Any typographical or grammatical errors should be corrected at revision, so please note any specific errors here.

Reviewer #1: Yes

5. Review Comments to the Author

Please use the space provided to explain your answers to the questions above. You may also include additional comments for the author, including concerns about dual publication, research ethics, or publication ethics. (Please upload your review as an attachment if it exceeds 20,000 characters)

Reviewer #1: The data availability statement did not indicate all data are included but has shared some and others are on the manuscript. The set of question asks for inclusion of all data and as such my response was no .

6. PLOS authors have the option to publish the peer review history of their article ([what does this mean?](https://journals.plos.org/globalpublichealth/s/editorial-and-peer-review-process#loc-peer-review-history)). If published, this will include your full peer review and any attached files.


**Do you want your identity to be public for this peer review?** If you choose “no”, your identity will remain anonymous but your review may still be made public.


For information about this choice, including consent withdrawal, please see our [Privacy Policy](https://www.plos.org/privacy-policy).

Reviewer #1: **Yes:** Nebreed Fesseha(MD)

[NOTE: If reviewer comments were submitted as an attachment file, they will be attached to this email and accessible via the submission site. Please log into your account, locate the manuscript record, and check for the action link "View Attachments". If this link does not appear, there are no attachment files.]

While revising your submission, please upload your figure files to the Preflight Analysis and Conversion Engine (PACE) digital diagnostic tool, <https://pacev2.apexcovantage.com/>. PACE helps ensure that figures meet PLOS requirements. To use PACE, you must first register as a user. Registration is free. Then, login and navigate to the UPLOAD tab, where you will find detailed instructions on how to use the tool. If you encounter any issues or have any questions when using PACE, please email PLOS at [figures@plos.org](mailto:figures@plos.org). Please note that Supporting Information files do not need this step.

References

1.	Rose S, Van Der Laan MJ. Why match? Investigating matched case-control study designs with causal effect estimation. Int J Biostat. 2009;5(1).

2.	Umezurike IS. Exploring the Adoption of Electronic Medical Records in Primary Health Care Centres in Calabar Municipality: The Challenges and Prospects of E-Governance. J Good Gov Sustain Dev Africa. 2021;6(2):70–80.

3.	Gardosi J, Kady SM, McGeown P, Francis A, Tonks A. Classification of stillbirth by relevant condition at death (ReCoDe): population based cohort study. Bmj. 2005;331(7525):1113–7.

4.	Geleto A, Chojenta C, Musa A, Loxton D. Barriers to access and utilization of emergency obstetric care at health facilities in sub-Saharan Africa: a systematic review of literature. Syst Rev. 2018;7(1):1–14.

5.	Singh S, Doyle P, Campbell OM, Mathew M, Murthy GVS. Referrals between public sector health institutions for women with obstetric high risk, complications, or emergencies in India–A systematic review. PLoS One. 2016;11(8):e0159793.

6.	Kasa GA, Woldemariam AY, Adella A, Alemu B. The factors associated with stillbirths among sub-saharan African deliveries: a systematic review and meta-analysis. BMC Pregnancy Childbirth. 2023;23(1):835.

7.	Wolde J, Haile D, Paulos K, Alemayehu M, Adeko AC, Ayza A. Prevalence of stillbirth and associated factors among deliveries attended in health facilities in Southern Ethiopia. PLoS One. 2022;17(12):e0276220.
